# Canonical binding of *Chaetomium thermophilum* DNA polymerase δ/ζ subunit PolD3 and flap endonuclease Fen1 to PCNA

**DOI:** 10.3389/fmolb.2023.1320648

**Published:** 2023-12-18

**Authors:** Magnus S. Alphey, Campbell B. Wolford, Stuart A. MacNeill

**Affiliations:** Biomedical Sciences Research Complex, School of Biology, University of St Andrews, St Andrews, United Kingdom

**Keywords:** PCNA, PIP motif, DNA replication, DNA polymerase δ, *Chaetomium thermophilum*

## Abstract

The sliding clamp PCNA is a key player in eukaryotic genome replication and stability, acting as a platform onto which components of the DNA replication and repair machinery are assembled. Interactions with PCNA are frequently mediated via a short protein sequence motif known as the PCNA-interacting protein (PIP) motif. Here we describe the binding mode of a PIP motif peptide derived from C-terminus of the PolD3 protein from the thermophilic ascomycete fungus *C. thermophilum*, a subunit of both DNA polymerase δ (Pol δ) and the translesion DNA synthesis polymerase Pol ζ, characterised by isothermal titration calorimetry (ITC) and protein X-ray crystallography. In sharp contrast to the previously determined structure of a *Chaetomium thermophilum* PolD4 peptide bound to PCNA, binding of the PolD3 peptide is strictly canonical, with the peptide adopting the anticipated 3_10_ helix structure, conserved Gln441 inserting into the so-called Q-pocket on PCNA, and Ile444 and Phe448 forming a two-fork plug that inserts into the hydrophobic surface pocket on PCNA. The binding affinity for the canonical PolD3 PIP-PCNA interaction determined by ITC is broadly similar to that previously determined for the non-canonical PolD4 PIP-PCNA interaction. In addition, we report the structure of a PIP peptide derived from the *C. thermophilum* Fen1 nuclease bound to PCNA. Like PolD3, Fen1 PIP peptide binding to PCNA is achieved by strictly canonical means. Taken together, these results add to an increasing body of information on how different proteins bind to PCNA, both within and across species.

## 1 Introduction

Highly efficient chromosomal DNA replication is essential for all forms of cellular life and requires the complex interplay of a wide range of protein factors in a temporally and spatially coordinated manner. The importance of high-fidelity chromosome replication is underlined by the fact that in humans, replication defects can lead to genetic disease (Weedon et al., 2013; Pelosini et al., 2014; Cui et al., 2020) and cancer (Church et al., 2013; Palles et al., 2013; Valle et al., 2014; Bellido et al., 2016; Rayner et al., 2016).

The sliding clamp PCNA (proliferating cell nuclear antigen) is a central player in multiple aspects of chromosomal DNA replication, repair and genome stability (Moldovan et al., 2007; Boehm et al., 2016). This ring-shaped homotrimer encircles double-stranded DNA to act as a processivity factor for DNA polymerases and a landing pad for the assembly of various DNA processing factors such DNA ligase I, the flap endonuclease Fen1, and the clamp loader complex replication factor C (RF-C) which opens and closes the PCNA ring around dsDNA. Many of the proteins that interact with PCNA, including DNA polymerase δ, DNA ligase I, Fen1 and RF-C, do so via a common mechanism that involves a short linear interaction motif on the partner protein called a PCNA-interacting protein (PIP) motif (Warbrick, 1998; Hamdan and De Biasio, 2023). The best characterised PIP motifs have conserved sequence Qxxψxxθθ, where ψ and θ represent amino acids with hydrophobic and aromatic side chains, respectively, but other variations on this sequence (so-called non-canonical PIP motifs) have been identified (Boehm and Washington, 2016; Prestel et al., 2019) such as ψxxxθ (Gonzalez-Magana et al., 2019) and Qxxψxθ (Yang et al., 2023).

DNA polymerase δ (Pol δ) plays a key role in chromosomal DNA replication and in multiple DNA repair processes (Guilliam and Yeeles, 2020). Pol δ is responsible for the bulk of Okazaki fragment synthesis on the lagging strand and is also involved in the initiation of leading strand synthesis at replication origins (Aria and Yeeles, 2018). Human Pol δ is a homotetramer, comprised of the catalytic subunit p125 (PolD1), p50 (PolD2), p66 (PolD3) and p12 (PolD4), whereas the well-studied budding yeast *Saccharomyces cerevisiae* Pol δ is a heterotrimer of the p125, p55 and p66 orthologues Pol3, Pol31 and Pol32, respectively (Lancey et al., 2020; Zheng et al., 2020). The p50 (PolD2, Pol31) and p66 (PolD3, Pol32) subunits of Pol δ are also found as components of the trans-lesion synthesis DNA polymerase Pol ζ, in complex with the Pol ζ catalytic subunit REV3L (yeast Rev3) and two copies of the accessory subunit REV7 (Rev7) (Malik et al., 2020).

Three of the four human Pol δ subunits contain PIP motifs that have been shown to bind PCNA: p125 (PolD1), p66 (PolD3) and p12 (PolD4). In the case of p66, the PIP motif is located at the extreme C-terminus of the protein at the end of lengthy region that is predicted to be largely unstructured and highly flexible (Reynolds et al., 2000; Johansson et al., 2004). Similarly, the PolD4 PIP motif is located at the end of an unstructured, flexible region close to the N-terminal end of the protein (Gonzalez-Magana et al., 2019; Yang et al., 2023). In the cryo-EM structure of human Pol δ bound to PCNA and DNA, only the catalytic subunit p125 (PolD1) PIP motif is seen interacting with PCNA (Lancey et al., 2020), suggesting that the PolD3 and PolD4 PIP motifs do not bind PCNA stably under these conditions. Similarly, in the cryo-EM structure of yeast Pol δ bound to PCNA and DNA, only the PolD1 orthologue Pol3 PIP motif binds to PCNA (Zheng et al., 2020).

Understanding how these various PIP motifs contribute to overall Pol δ function requires a combination of functional and structural analysis. In an effort to gain mechanistic insights into Pol δ function, we have developed tools for high-level expression and rapid purification of the four-subunit Pol δ complex encoded by the thermophilic ascomycete fungus *Chaetomium thermophilum* (*Ct*, also known as *Thermochaetoides thermophila*) (D. Yang and S. MacNeill, unpublished). In parallel with this, we have embarked on a study of how *Ct* Pol δ interacts with *Ct* PCNA and have previously described the non-canonical binding of the N-terminal PIP motif from the *Ct* PolD4 protein to *Ct* PCNA (Yang et al., 2023). Here, we report characterisation of the interaction between the *Ct* PolD3 PIP motif and its cognate PCNA by protein X-ray crystallography at a resolution of 2.45 Å. Unlike the *Ct* PolD4-*Ct* PCNA interaction, *Ct* PolD3 binding to *Ct* PCNA is in every respect canonical: Gln441 inserts into the Q-pocket, a 3_10_ helix is formed and Ile444 and Phe448 form the two-fork plug. Interestingly, despite this canonical binding mode, the affinity of the PIP motif peptide for *Ct* PCNA, determined by isothermal titration calorimetry (ITC), is approximately two-fold lower than that seen with the non-canonical binding of the *Ct* PolD4 PIP to *Ct* PCNA (43 µM *versus* 22 µM). In addition to this, we present the X-ray crystal structure of the *Ct* Fen1 nuclease PIP peptide bound to *Ct* PCNA at 1.95Å. This too displays canonical binding to *Ct* PCNA, with the 3_10_ helix-containing peptide engaging in Q-pocket and two-fork plug interactions. We discuss these results in terms of the conservation and divergence of PIP motif sequences both within and across species.

## 2 Materials and methods

### 2.1 Identification of *Ct* PolD3 and *Ct* Fen1 proteins

Sequences encoding *Ct* PolD3 and *Ct* Fen1 were identified by BLASTP searching the nr database with the ascomycete *Schizosaccharomyces pombe* Cdc27 (PolD3) and Rad2 (Fen1) protein sequences as the queries (UniProt accession numbers P30261 and P39750, respectively). As protein sequence comparisons suggested that the *Ct* PolD3 protein sequence predicted in the database was N-terminally truncated, full-length cDNAs encoding PolD3 were subsequently amplified from *C. thermophilum* DSM 1495 cDNA (a generous gift of E. Hurt, University of Heidelberg) and sequenced, allowing identification of the complete ORF. The full-length cDNA sequence has been deposited with the GenBank database (accession number OQ605904) and the full-length protein sequence with UniProt (accession number G0S636). The *Ct* Fen1 protein has accession number G0S2B5 in the UniProt database. Protein sequence alignments for PolD3 and Fen1 can be seen as Supplementary Figures S1, S2, respectively.

### 2.2 Protein expression and purification

*Ct* PCNA was expressed and purified to apparent homogeneity as described previously (Yang et al., 2023). Briefly, the protein was expressed in recombinant form in *E. coli* with a TEV protease-cleavable N-terminal His6 tag, purified using IMAC, cleaved with His6-TEV to remove the His6 tag, subjected to reverse IMAC to remove still-tagged PCNA protein and His6-TEV protease, then polished using SEC. Purified protein (11.0 mg/mL) was flash-frozen in liquid nitrogen and stored at −80°C.

### 2.3 Peptides

A 15mer *Ct* PolD3 PIP peptide spanning residues 437–451 (sequence: ^437^GKGGQGSIMSWFAKK^451^) and a 15mer *Ct* Fen1 peptide spanning residues 339–353 (^339^GAQQARIEGFFKVIP^353^) were commercially synthesised (GenScript, Piscataway, New Jersey, United States) and obtained in lyophilised form at final purities of 99.4% and 99.1%, respectively. The *Ct* PolD3 peptide was resuspended in either dH_2_O or 50 mM Tris-HCl, 50 mM NaCl, pH 8.0 at a concentration of 8 mg/mL (5.1 mM), while the *Ct* Fen1 peptide (insoluble in aqueous solution) was resuspended in DMSO at a concentration of 8 mg/mL (4.8 mM).

### 2.4 Crystallography

For crystal screens, 90 µL of 14 mg/mL *Ct* PCNA was mixed with 21 µL of either 8 mg/mL *Ct* PolD3 PIP peptide (in dH_2_O) or 8 mg/mL *Ct* Fen1 PIP peptide (in DMSO) and screened using JCSG Plus™ and PACT Premier™ screens (Molecular Dimensions, Holland, OH, United States). Diffraction quality crystals were obtained from the JCSG Plus™ screen in 0.1 M phosphate/citrate pH 4.2, 40% PEG 300 (for *Ct* PolD3 peptide-*Ct* PCNA) and 2.4 M sodium malonate dibasic monohydrate pH 7.0 (for *Ct* Fen1 peptide-*Ct* PCNA). The crystals diffracted to 2.45 and 1.95 A, respectively. The data for both complexes were collected in-house at 100 K on a Rigaku MM007HF Cu anode X-ray generator (Rigaku, Tokyo, Japan). Reflections were recorded on a Rigaku Saturn 944+ CCD detector. Data processing was performed using iMOSFLM (Battye et al., 2011), scaled using AIMLESS, and the space group of each structure was identified using POINTLESS (Murshudov et al., 2011). *Ct* PCNA-*Ct* PolD3 was crystallized in space group P1 with one trimer of *Ct* PCNA in the asymmetric unit, while *Ct* PCNA-*Ct* Fen1 was crystallized in space group H32 with one chain of *Ct* PCNA in the asymmetric unit. The data collection statistics are found in Supplementary Table S1. The structures of the protein-peptide complexes were solved by molecular replacement using MOLREP (CCP4) (Vagin and Teplyakov, 1997) using the complete structure of *Ct* PCNA (PDB: 7O1E) as a starting model (Yang et al., 2023). Repeated rounds of model refinement using COOT (Emsley and Cowtan, 2004) and REFMAC5 (Murshudov et al., 2011) resulted in a structural model with an R_work_ of 21.2% and R_free_ of 24.9% for *Ct* PCNA-PolD3 PIP and R_work_ of 17.1% and R_free_ of 20.4% for *Ct* PCNA-Fen1 PIP (see Supplementary Table S1). The structures have been deposited in the PDB with accession codes 8P9O (*Ct* PCNA-*Ct* PolD3 PIP) and 8Q7I (*Ct* PCNA-*Ct* Fen1 PIP).

### 2.5 Isothermal titration calorimetry (ITC)

ITC was performed using a MicroCal PEAQ-ITC calorimeter (Malvern Panalytical, Malvern, UK). Prior to measurement, the *Ct* PolD3 peptide and *Ct* PCNA protein were buffer-exchanged into 50 mM Tris-HCl, 150 mM NaCl, pH 8.0. Experimental titrations were performed at 25°C in duplicate with 300 µL of 32.7 µM *Ct* PCNA and 420 µM *Ct* PolD3 peptide. Control titrations used *Ct* PolD3 peptide only at 420 µM. In total, 19 injections were used for each assay: a primary injection of 0.2 µL followed by 18 injections of 2.0 µL. The heat change following injection was measured, the control values subtracted, and data was fitted to a single site model (1 peptide: 1 PCNA protomer) using MicroCal PEAQ-ITC Analysis Software (v1.21). The K_*D*_ values reported represent the mean of the two experiments.

## 3 Results

### 3.1 *Chaetomium thermophilum* PolD3 protein

**FIGURE 1:**
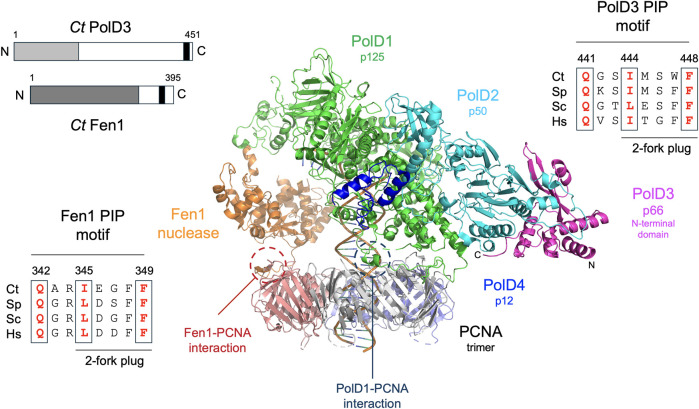
Overall structure of Pol δ and conservation of PolD3 and Fen1 PIP motif sequences. Centre: Cryo-EM structure of human Pol δ-Fen1-PCNA-DNA complex determined by De Biasio and coworkers (Lancey et al., 2020). Individual proteins are shown in different colours as follows: PolD1/p125, green; PolD2/p50, cyan; PolD3/p66 N-terminal domain, magenta; PolD4/p12 C-terminal domain, dark blue; Fen1, orange; PCNA promoters, grey, purple and salmon pink. The N- and C-terminal ends of PolD3 are labelled N and C. PCNA-PIP peptide interactions involving Fen1 and PolD1/p125 are circled. Top left: Schematic representation of *Ct* PolD3 and *Ct* Fen1 proteins, with the PolD3 N-terminal domain shown in light grey, the Fen1 nuclease domain in dark gray, and the two PIP motifs in black. Top right: Protein sequence alignment of known or predicted PIP motifs in PolD3 proteins from *Chaetomium thermophilum* (Ct), *S. pombe* (Sp), *S. cerevisiae* (Sp) and human (Hs). Bottom left: Protein sequence alignment of PIP motifs in Fen1 proteins from *Chaetomium thermophilum* (Ct), *S. pombe* (Sp), *S. cerevisiae* (Sc) and human (Hs). Key conserved residues are shown in bold red. Central image prepared using the PyMOL Molecular Graphics System version 2.0 (Schrödinger LLC, New York) and PDB file 6TNZ.

BLASTP searching using the ascomycete fission yeast *S. pombe* PolD3 orthologue Cdc27 as the query sequence led (after later cDNA sequencing, see Materials and methods for details) to the identification of *Ct* PolD3 as a 451 amino acid protein with predicted molecular weight 49.3 kDa. *Ct* PolD3 is 26% identical to *S. pombe* Cdc27 at the amino acid sequence level and ∼20% identical to the human and *S. cerevisiae* PolD3 orthologues p66 and Pol32, respectively. The conserved PIP motif found at the extreme C-terminus of human, *S. cerevisiae* and *S. pombe* PolD3 orthologues is readily identifiable at the C-terminal end of the *Ct* PolD3 protein (sequence: ^441^ QGSIMSWF ^448^ conserved PIP motif residues underlined) (Figure 1, Supplementary Figure S1).

### 3.2 Binding of *Ct* PolD3 PIP peptide to PCNA

**FIGURE 2:**
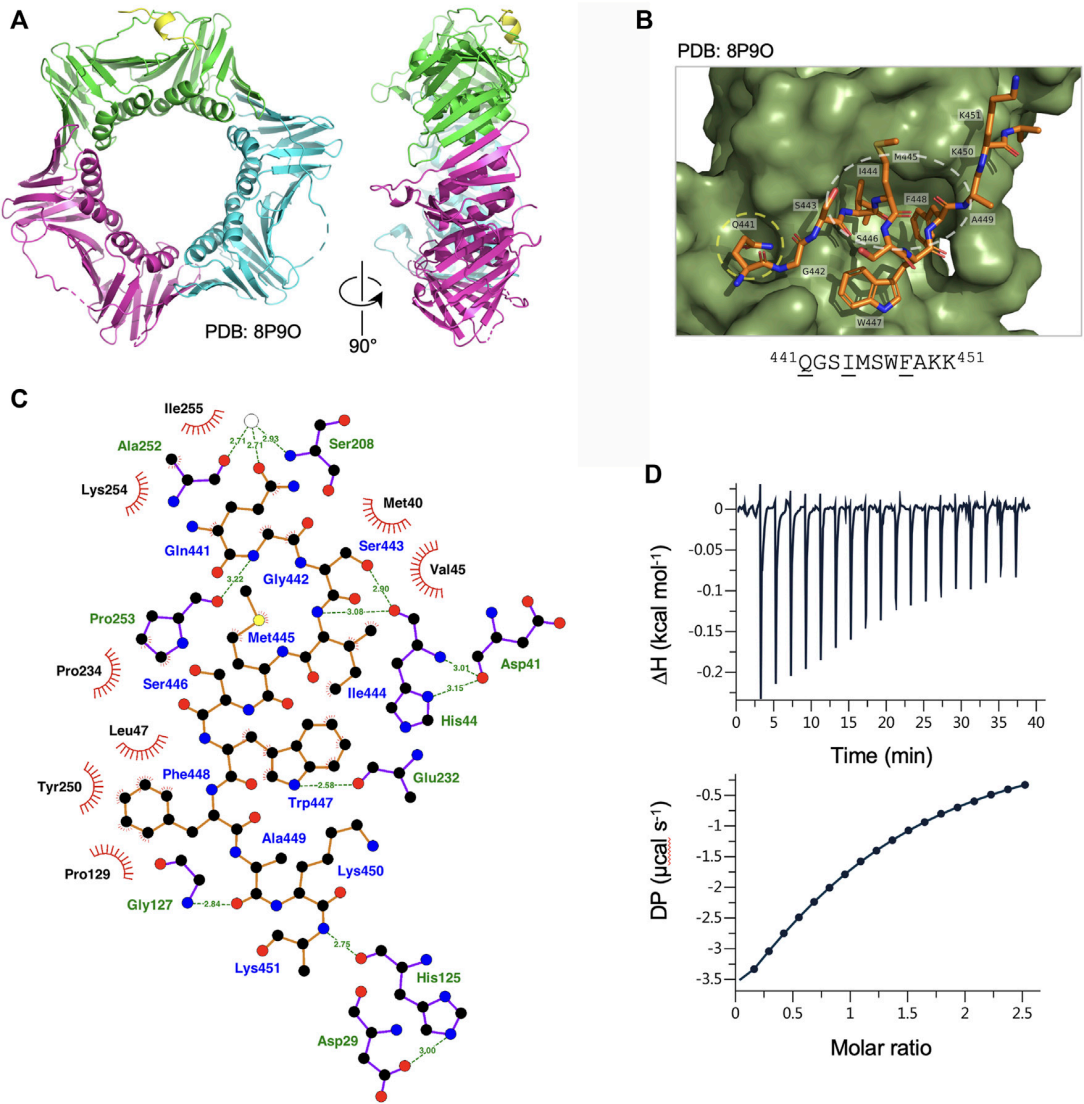
Binding of Ct PolD3 PIP peptide to Ct PCNA. **(A)** Crystal structure of *Ct* PCNA (shown in green, cyan and magenta) with a *Ct* PolD3 PIP peptide (yellow) binding to one of the PCNA protomers at 2.45 Å resolution (PDB: 8P9O). **(B)** Close-up view of the *Ct* PolD3 PIP peptide (residues visible: ^441^ QGSIMSWFAKK^451^, with conserved PIP motif residues underlined) bound to *Ct* PCNA. The Q-pocket on *Ct* PCNA (occupied by the sidechain of Gln441) is circled in yellow and the hydrophobic surface pocket (occupied by the sidechains of Ile444 and Phe448) in white. Image prepared using the PyMOL Molecular Graphics System version 2.0 (Schrödinger LLC, New York) and PDB file 8P9O. **(C)** Detailed view of the *Ct* PCNA-*Ct* PolD3 PIP peptide interaction represented using LigPlot + version 2.2.5 (Laskowski and Swindells, 2011) with default parameters. The *Ct* PolD3 peptide backbone is shown in orange, with individual peptide amino acids labelled in blue text. Hydrogen bonds (2.7–3.35 Å) are shown as green broken lines, with bond distances (Å) indicated. Amino acids in *Ct* PCNA involved in hydrogen bonding to the *Ct* PolD3 PIP peptide are labelled in green text. The red spoked arcs represent residues in *Ct* PCNA making hydrophobic contacts (2.9–3.9 Å) with the peptide; the corresponding atoms in the *Ct* PolD3 peptide are decorated with smaller red spokes. Carbon, oxygen, nitrogen and sulphur atoms are represented by black, red, dark blue and yellow circles, respectively, and water molecules by white circles. **(D)** Affinity of *Ct* PCNA for the *Ct* PolD3 peptide measured by isothermal titration calorimetry (ITC). The left-hand panel shows baseline-corrected experimental data from titration of the *Ct* PolD3 peptide (420 µM) with *Ct* PCNA (32.7 µM). The right-hand panel shows the ligand concentration dependence of heat released upon PCNA-peptide binding, with the molar ratio referring to peptide:PCNA promoter. The ITC analysis was performed twice with similar results; a single dataset is shown. See Materials and methods for details.

To characterise the binding of the *Ct* PolD3 with *Ct* PCNA, the structure of the *Ct* PolD3 PIP motif peptide-*Ct* PCNA complex was solved by X-ray crystallography at a resolution of 2.45 Å (Figure 2A, see Supplementary Table S1 for crystallography statistics and Supplementary Figure S3 for Fo−Fc difference density maps). The *Ct* PolD3 peptide (sequence: ^437^GKGGQGSIMSWFAKK^451^ with conserved PIP motif residues underlined) occupied a single PIP binding site only, with the other sites on *Ct* PCNA being occluded by crystal packing (specifically, by the loop that spans residues Thr186-Lys190 in neighbouring symmetry mates). Only 11 of 15 residues in the peptide could be seen in the electron density map (Gln441 to Lys451) suggesting that the N-terminal four residues of the peptide are flexible (Figure 2B).

Consistent with the high degree of conservation of the PIP peptide sequence at the C-terminal end of PolD3 orthologues, the mode of PCNA-PIP binding observed is strictly canonical (Figure 2B): part of the peptide adopts a 3_10_ helical structure (Figure 2A), *Ct* PolD3 Gln441 inserts into the Q-pocket on *Ct* PCNA and *Ct* PolD3 Ile444 and Phe448 form a 2-fork plug that inserts into the hydrophobic surface pocket on *Ct* PCNA (Figure 2B). Both hydrogen bonding and hydrophobic interactions are apparent (shown in detail in Figure 2C). *Ct* PolD3 Gln441(OE1) makes water-mediated hydrogen bonding interactions with Ser208 (N) and Ala252 (O) on *Ct* PCNA, Gly442 (N) hydrogen bonds with Pro253 (O), Ser 443 (OG) and Ile444 (N) both hydrogen bond with His44(O), Trp447 (NE1) hydrogen bonds with Glu232 (O), Ala449 (O) with Gly127 (N), and Lys451 (N) with His125 (O). Hydrophobic interactions with *Ct* PCNA are seen with *Ct* PolD3 Gln441, Gly442, Ile444, Met445, Trp447 and Phe448. Interactions involving Ile444 (specifically involving CB, CG, CD1, CZ2, CZ3) and Phe448 (CD1, CD2, CE1, CE2, CZ) anchor the 2-fork plug in the hydrophobic surface pocket formed by Leu47, Pro129, Pro234 and Pro250 in *Ct* PCNA (Figure 2C).

### 3.3 Binding affinity determined by isothermal titration calorimetry

In order to gauge the affinity of the *Ct* PolD3 PIP peptide for *Ct* PCNA, isothermal titration calorimetry (ITC) was used to determine the binding affinity of *Ct* PCNA for the 15mer *Ct* PolD3 PIP motif peptide (^437^GKGGQGSIMSWFAKK^451^) and the stoichiometry of the interaction in solution (Figure 2D). The mean dissociation constant (K_*D*_) from two experiments was determined to be 43.2 µM ± 3.9 μM at 25°C and the stoichiometry 1:1 (i.e. 1 PIP peptide: 1 PCNA protomer) indicating that all three PIP peptide binding sites on *Ct* PCNA are occupied in solution, as expected. The measured K_*D*_ is almost three-fold less (43 µM *versus* 16 µM) than that previously reported for the human PolD3/p66-PCNA interaction determined by ITC with a peptide of sequence ^452^KANRQVSITGFFQRK^466^ (Bruning and Shamoo, 2004) and two-fold less (43 µM *versus* 22 µM) than that for *Ct* PolD4 binding to *Ct* PCNA despite the latter interaction involving a non-canonical binding mode (Yang et al., 2023). This is discussed further below (see Discussion).

### 3.4 Binding of *Ct* Fen1 PIP peptide to *Ct* PCNA

Fen1 is a 5’ flap-specific nuclease that plays an important in Okazaki fragment maturation. BLASTP searching with the *S. pombe* Fen1 nuclease orthologue Rad2 identifies *C. thermophilum* Fen1 as a 395 amino acid protein that is 60% identical to the *S. pombe* and *S. cerevisiae* Fen1 orthologues Rad27 and Rad2, respectively, at the amino acid sequence level and 55% identical to human Fen1. Key catalytic residues in human Fen1 are conserved in the *C. thermophilum* enzyme, as is the PIP motif located towards the C-terminal end of the protein (sequence: ^342^ QARIEGFF ^349^ with conserved PIP motif residues underlined) (Figure 1).

**FIGURE 3:**
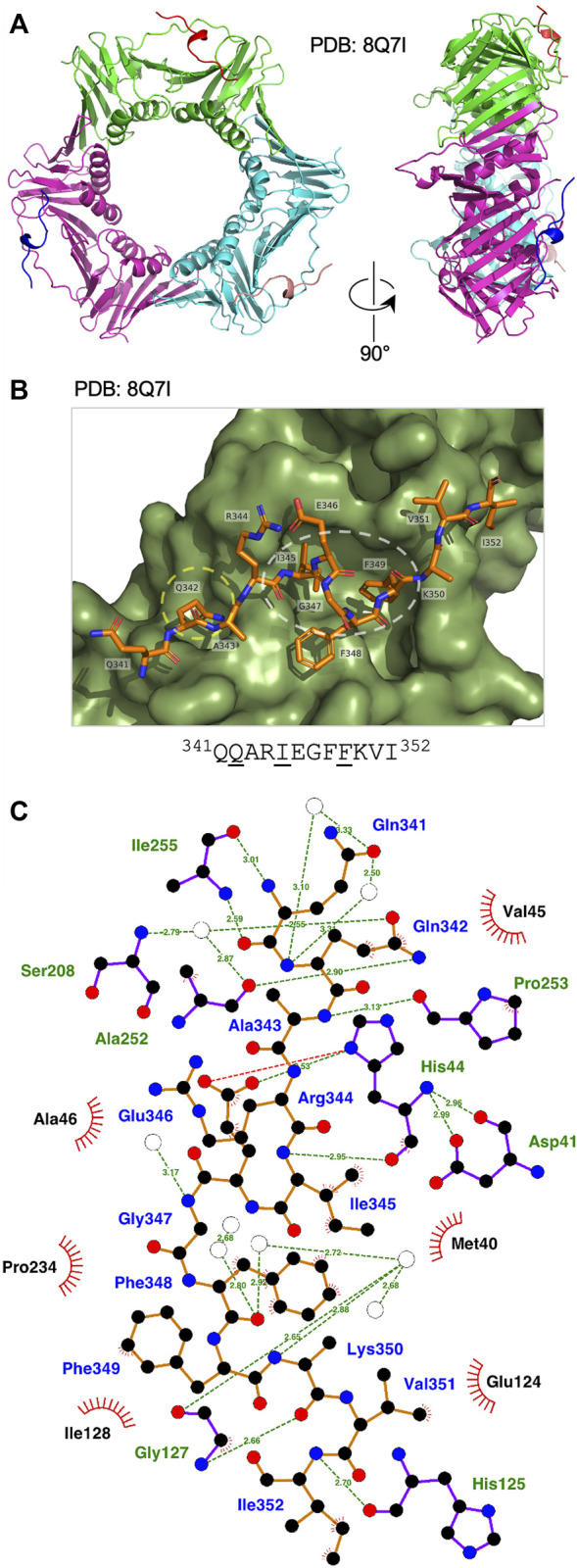
Binding of Ct Fen1 PIP peptide to Ct PCNA. **(A)** Crystal structure of *Ct* PCNA (shown in green, cyan and magenta) bound to the *Ct* Fen1 PIP peptides (red, salmon pink, blue) at 1.95 Å resolution (PDB: 8Q7I). **(B)** Close-up view of the *Ct* Fen1 PIP peptide (residues visible: ^341^QQARIEGFFKVI^352^, with conserved PIP motif residues underlined) bound to *Ct* PCNA. The Q-pocket on *Ct* PCNA (occupied by the sidechain of Gln342) is circled in yellow and the hydrophobic surface pocket (occupied by the sidechains of Ile345 and Phe349) in white. Image prepared using the PyMOL Molecular Graphics System version 2.0 (Schrödinger LLC, New York) and PDB file 8Q7I. **(C)** Detailed view of the *Ct* PCNA-*Ct* Fen1 PIP peptide interaction represented using LigPlot + version 2.2.5 (Laskowski and Swindells, 2011) with default parameters. The *Ct* Fen1 peptide backbone is shown in orange, with individual peptide amino acids labelled in blue text. Hydrogen bonds (2.7–3.35 Å) are shown as green broken lines, with bond distances (Å) indicated. Amino acids in *Ct* PCNA involved in hydrogen bonding to the *Ct* Fen1 PIP peptide are labelled in green text. The red spoked arcs represent residues in *Ct* PCNA making hydrophobic contacts (2.9–3.9 Å) with the peptide; the corresponding atoms in the *Ct* Fen1 peptide are decorated with smaller red spokes. Carbon, oxygen, nitrogen and sulphur atoms are represented by black, red, dark blue and yellow circles, respectively, and water molecules by white circles.

The *Ct* Fen1 PIP peptide-*Ct* PCNA structure was solved to a resolution of 1.95 Å (Figure 3A, see also Supplementary Table S1, Supplementary Figure S3). 14 of 15 residues in the *Ct* Fen1 PIP peptide were visible in the structure, revealing a 3_10_ helix and the anticipated canonical binding mode, with *Ct* Fen1 Gln342 inserting into the Q-pocket, and Ile345 and Phe349 forming the two-fork plug that inserts into the hydrophobic surface pocket on *Ct* PCNA (Figure 3B). As with *Ct* PolD3, the *Ct* Fen1 Q-pocket glutamine, Gln342, interacts with Ser208 and Ala252 in *Ct* PCNA, with the interaction with Ser208 involving water-mediated hydrogen bonding between Gln342 (OE1) and Ser208 (N), as is the case for the equivalent Gln441-Ser208 pairing in *Ct* PolD3, whereas the interaction with Ala252 involves a direct H-bond between Gln342 (NE2) and Ala252 (O), different from the water-mediated Gln441-Ala252 interaction in the *Ct* PolD3-*Ct* PCNA structure. Additional direct hydrogen-bonding interactions are seen involving Gln341 (N) and Ile255 (O), Gln341 (O) and Ile255 (N), Ala343 (N) and Pro253 (O), Ile345 (N) and His44 (O), Glu346 (OE2) and His44 (ND1), Lys350 (O) and Gly127 (N), and Ile353 (N) and His125 (O). In addition to this, the higher resolution of the structure allowed identification of multiple water-mediated hydrogen bonds connecting the *Ct* Fen1 peptide to *Ct* PCNA (Figure 3C). A salt bridge links Glu346 (OE1) and His44 (ND1) also. As with the *Ct* PolD3-*Ct* PCNA structure, the 2-fork plus residues Ile345 and Phe349 display hydrophobic interactions with the surface pocket on *Ct* PCNA (Figure 3C) although in the case of Phe349 only a single interaction (involving Phe349 (CE1)) is apparent within the 3.9 Å length cut-off applied in the analysis.

## 4 Discussion

**FIGURE 4:**
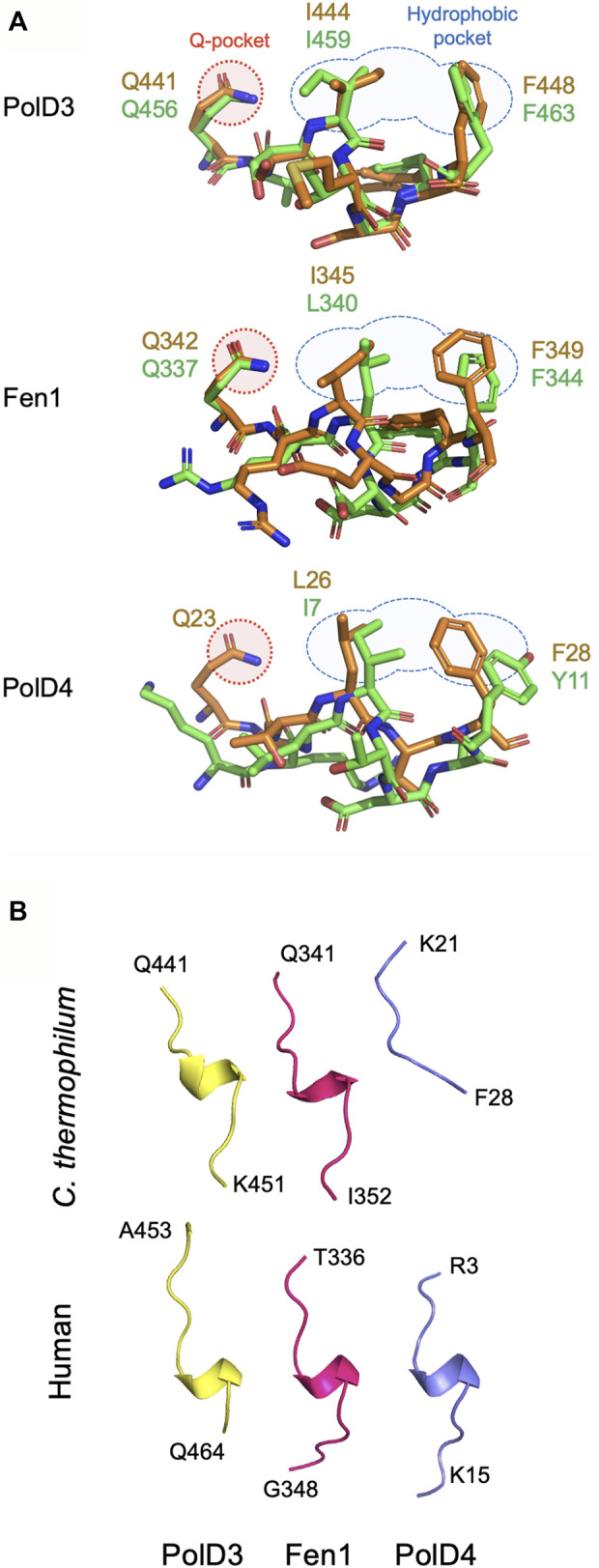
Comparison of PIP peptide binding modes. **(A)** Overlaid *Chaetomium thermophilum* and human PIP motif peptide structures from PolD3 (upper panel), Fen1 (middle panel) and PolD4 (lower panel) with structures and key residues from *Chaetomium thermophilum* and human shown in orange and green, respectively. PDB codes: 8P9O (*Ct* PolD3), 1U76 (human PolD3/p66), 8Q7I (*Ct* Fen1), 1U7B (human Fen1), 7O1F (*Ct* PolD4) and 6HVO (human PolD4/p12). The Q-pocket and hydrophobic pocket are shown in outline in red and blue, respectively. **(B)** Ribbon structures of the six PIP motif peptides shown above highlighting the presence of a 3_10_ helix in all but the *Ct* PolD4 PIP peptide. Images prepared using the PyMOL Molecular Graphics System version 2.0 (Schrödinger LLC, New York).

Beginning with the structure of p21^Cip1^ PIP bound to PCNA (Gulbis et al., 1996), the atomic structures of a large and diverse set of PCNA-PIP peptide complexes have been determined over the last quarter century (Prestel et al., 2019), although only in very few cases have equivalent (orthologous) interactions been studied in multiple species. Here we present atomic structures of PCNA-PIP peptide complexes derived from the *Ct* PolD3 and *Ct* Fen1 proteins bound to their cognate PCNA at resolutions of 2.45 and 1.95 Å, respectively. In both cases, the new structures allow comparison with earlier structures of human orthologues: *Ct* PolD3-*Ct* PCNA with human PolD3/p66-PCNA and *Ct* Fen1-*Ct* PCNA with human Fen1-PCNA (Bruning and Shamoo, 2004) (Figure 4).

In sharp contrast to the non-canonical *Ct* PolD4-*Ct* PCNA and human p12-PCNA interactions described previously, the *Ct* PolD3-*Ct* PCNA and *Ct* Fen1-*Ct* PCNA interactions described here are strictly canonical in nature (Gonzalez-Magana et al., 2019; Yang et al., 2023). Both PIP peptides form a 3_10_ helix, conserved glutamines (Gln441 and Gln342 in *Ct* PolD3 and *Ct* Fen1, respectively) insert into the *Ct* PCNA Q-pocket, and in both cases a 2-fork plug (involving the sidechains of Ile444 and F448 in *Ct* PolD3, and Leu345 and Phe349 in *Ct* Fen1) inserts into the hydrophobic pocket on the surface of *Ct* PCNA. Figure 4 shows a comparison of the binding modes of PolD3, Fen1 and PolD4 PIP peptides across species, highlighting the high level of cross-species similarity between the human and *C. thermophilum* PolD3-PCNA and Fen1-PCNA structures. This is in contrast to the more divergent PolD4 orthologue PIP-PCNA structures, where the *Ct* PolD4 PIP peptide does not form the 3_10_ helix characteristic of most other PIP peptides (including human PolD4/p12, but also *Ct* PolD3 and *Ct* Fen1), nor does the *Ct* PolD4-*Ct* PCNA interaction involve a glutamine-Q-pocket interaction (unlike human PolD4/p12, *Ct* PolD3 and *Ct* Fen1) (Figure 4) (Yang et al., 2023).

Interestingly, the binding affinities of the human and *C. thermophilum* PolD3 and PolD4 peptides for their cognate PCNAs determined by ITC are broadly similar, with measured K_*D*_ values ranging from 16 to 43 µM (Bruning and Shamoo, 2004; Gonzalez-Magana et al., 2019; Yang et al., 2023) and the differences that exist show no obvious pattern: in humans, the measured K_*D*_ for the canonical PolD3/p66-PCNA interaction is lower than that for the non-canonical PolD4-PCNA interaction (16 µM *versus* 38 µM) while in *C. thermophilum* the measured K_D_ for canonical PolD3-PCNA is higher than that for non-canonical PolD4-PCNA (43 µM *versus* 22 µM), suggesting that specific binding affinity for PCNA (within the observed 16–43 µM range) may not be crucial for PolD3 and PolD4 protein function.

Remarkably, the PolD3-PCNA interaction is not seen in recent cryo-EM structures of human or budding yeast Pol δ complexed with PCNA on primer-template DNA (Zheng et al., 2020; Lancey et al., 2021). The human PolD4 PIP-PCNA interaction is also not seen. In both sets of Pol δ-PCNA structures, the only visible PIP motif-PCNA interaction is that between the catalytic subunit of Pol δ (PolD1/human p125/yeast Pol3) and PCNA, with the two remaining PIP peptide binding sites on the PCNA trimer being unoccupied. These sites are not occluded however, as evidenced by Fen1-PCNA interaction in the human Pol δ-PCNA-Fen1 complex (shown in Figure 1) (Lancey et al., 2021). Why these interactions are not seen remains unclear but it is possible that they have only a very limited role, or no role at all, to play once the Pol δ complex has engaged with its substrate at the primer-template junction. Instead, the PolD3 and PolD4 PIP motifs might facilitate recruitment of Pol δ to PCNA that has previously been loaded at the primer-template junction, before handing over the task of stabilizing Pol δ-PCNA interaction to PolD1 PIP-PCNA binding, or might ensure that should Pol δ disengage from the primer-template, it is retained in the vicinity of the DNA to allow efficient polymerase recycling. The PolD3 and PolD4 PIP motifs are located at the end of lengthy flexible regions that could, in either case, be employed in the “fly casting mechanism” (Shoemaker et al., 2000) to scan three-dimensional space and latch onto PCNA prior to the Pol δ-PCNA complex being locked more securely into position via the PolD1 PIP-PCNA interaction seen in the cryo-EM structures (Hamdan and De Biasio, 2023). A similar mechanism has been proposed for DNA ligase I (Lig1)-PCNA interactions: a PIP motif at the N-terminal end of the flexible N-terminal region of Lig1 (PIP_N-term_) tethers the protein to PCNA when the ligase is detached from DNA but once the ligase locates a nick, this interaction is disrupted and a second PIP motif (PIP_DBD_), located near the centre of the Lig1 DNA binding domain, engages with PCNA instead (Blair et al., 2022).

A key functional distinction between the human PolD3/p66 and PolD4/p12 PIP motifs is that the latter is PIP degron, a specialized PIP motif that acts as a targeting signal for protein degradation (Havens and Walter, 2009; Havens and Walter, 2011; Havens et al., 2012). Once bound to PCNA on chromatin, PIP degron-containing proteins such as PolD4/p12, Cdt1 and p21^Cip1^ are ubiquitylated and degraded by a CRL^Cdt2^-dependent mechanism. Degradation of human PolD4/p12 occurs as cells enter S-phase or in response to DNA damage (Zhang et al., 2007; Meng et al., 2009; Terai et al., 2013; Zhang et al., 2013), leaving behind a three-subunit Pol δ complex that appears better suited to the task in hand (Lee et al., 2014; Lee et al., 2019). It remains to be seen whether the *Ct* PolD4 protein is degraded via a similar mechanism, however, we have shown that the orthologous PolD4/Cdm1 protein from the related ascomycete fission yeast *S. pombe* is a CRL^Cdt2^ substrate (S.M., unpublished results) suggesting that *Ct* PolD4 protein levels may be regulated in this way too. Previously, a basic amino acid four residues C-terminal to the 2-fork plug aromatic residue in the conserved PIP motif sequence (i.e., in the +4 position) has been identified as being an important (though not defining) feature of PIP degrons (Havens and Walter, 2009; Havens and Walter, 2011; Havens et al., 2012); filamentous fungal PolD4 proteins such as *Ct* PolD4 lack this, but do have conserved basic residues at +3 and +6 that could play a similar role (Yang et al., 2023). In *S. pombe* and other fission yeasts, an arginine is found conserved at position +5. Neither PolD3 nor Fen1 is thought to be a CRL^Cdt2^ substrate, suggesting that instead of being related to PCNA binding affinity, the divergence of the PolD4 PIP-PCNA binding mode from canonical to non-canonical may be related to the targeting of these proteins by CRL^Cdt2^, their ubiquitylation and subsequent degradation. Further work on diverse PIP- and PIP degron-containing proteins will be required to address this.

In summary, the determination of the structures of *Ct* PolD3, PolD4 and Fen1 PIP peptides bound to *Ct* PCNA allows direct comparison of binding modes, both within species (PolD3 *versus* PolD4 *versus* Fen1) but also across species, with reference to previously determined structures for human PolD3/p66, PolD4/p12 and Fen1 PIP peptide-PCNA complexes (Bruning and Shamoo, 2004; Gonzalez-Magana et al., 2019). With increasing interest in the development of inhibitors of PCNA-PIP interactions for therapeutic purposes (Horsfall et al., 2020; Gu et al., 2023), gaining a detailed structural understanding of how these interactions occur, how they have evolved and how this impacts function, is more important than ever.

## Data Availability

The datasets presented in this study can be found in online repositories. The names of the repository/repositories and accession number(s) can be found below: PDB entry 8P9O: https://doi.org/10.2210/pdb8P9O/pdb; PDB entry 8Q7I: https://doi.org/10.2210/pdb8Q7I/pdb. The GenBank and UniProt database entries (OQ605904 and G0S636 respectively) are as follows: GenBank OQ605904: https://www.ncbi.nlm.nih.gov/nuccore/OQ605904; UniProt G0S636: https://www.uniprot.org/uniprotkb/G0S636/entry.
