# Supplementary material for: Canonical binding of Chaetomium thermophilum DNA polymerase δ/ζ subunit PolD3 and flap endonuclease Fen1 to PCNA
Source: Front Mol Biosci. 2023 Dec 18;10:1320648. doi: 10.3389/fmolb.2023.1320648 (PMC10787639; doi:10.3389/fmolb.2023.1320648)
Supplement: Supplementary file 1 [file DataSheet1.PDF]

	PCNA+PolD3	PCNA+Fen1
**PDB ID**	8P9O	8Q7I
**Data Collection**		
Space group	P1	H32
**Cell dimensions**		
a,b,c (Å)	55.84, 56.39, 62.86	87.35, 87.35, 214.33
α,β,γ (°)	90.93, 90.55, 99.48	90.00, 90.00, 120.00
Resolution (Å)	27.57 - 2.45	21.86 - 1.95
R_{merge}	0.091 (0.256)	0.07 (0.18)
I/σI	5.7 (2.8)	23.8 (11.1)
Completeness (%)	93.2 (91.0)	99.9 (99.7)
Redundancy	2.6 (2.4)	10.6 (8.6)
CC ½	0.988 (0.881)	0.999 (0.988)
**B-factors**		
Overall	32.7	17.8
Protein	32.8	16.3
Peptide	34.3	24.5
**Refinement**		
Resolution (Å)	27.57 – 2.45	21.86 - 1.95
No. reflections	24703	22067
R_{work}/R_{free} (%)	21.2 / 24.9	17.1 / 20.4
**No. of atoms**		
Protein	5617	1982
Peptide / ion	85 / 0	97 / 2
Water	152	280
**Ramachandran**		
Favoured (%)	98	99
Allowed (%)	2	1
Outliers (%)	0	0

Supplementary Table S1: Crystallography statistics. Numbers in parentheses are from the highest resolution bin.

```
Sc      -MDQKASYFINEKLFTEVKPVLFTDLIHHLKIGPSMAKKLMFDYYKQTTNAKYNCVVICC      59
Hs      MADQLYLENIDEFVTDQNKIVTYKWLSYTLGVHVNQAKQMLYDYVERKRKENSGAQLHVT      60
Ct      --MDCYTKYLAENVLSEDKVVTYRYLSRALRVHVNTAKQMLFEFHRSQNA-KCPNTVHAT      57
Sp      --MEEWRNFLDIKVINESSLVTVDNLSLQLDISSEKAQEYLNMFYQGNDF------LYPI      52
           :     :   :  : . *    *   * :  . *:: :  : .          :

Sc      YKDQTIKIIHDLS---------------NIPQQD---SIIDCFIYAFNPMDS--------      93
Hs      YLVSGSLIQNGHS----------------------------CHKVAVVREDKLEAVKSKL      92
Ct      YLVYGRKKADAEQPSALKNGDGDIEMTSSPPEAESISEAVPAYSLSLIPEDRLSDALADY      117
Sp      YLIHGQPIDDEIN---LE-----IDEESQP-----I-SNFPVLQYILCDKSSLQEKQSRL      98
        *        . .                                 .   .

Sc      ------------------FIPY----YDIIDQ------KDCLTIKNSYELKV--------      117
Hs      AVTASIHVYSIQKAMLKDSGPLFNTDYDILKSN-L---QNCSKFSAIQCAAAVPRAPAES      148
Ct      DEVFSIHVYSIGPHPNKDVALLADAANAT---LNLESKGDVTQLRAIINPRARRREQQGA      174
Sp      KSGYKTVIFALSSAPLSDFDELLPAVYEIREKDVLYKKEDADKYGFIFNENSV-------      151
                                               :

Sc      SESSKIIERT---KTLEEKSKPLVRPTA-------RSKTTPEET----------------      151
Hs      SSSSKKFEQSHLHMSSETQANNELTTNGHGPPASKQVSQQPKGIMGMFASKAAAKTQETN      208
Ct      G-----LRAAAAAATVKSQAKSIF-PKPPTAPSTSQVKEAPKAS----------QTVEET      218
Sp      --------------------PRVL-KKAPSTHSP-Q-LSVPSKT----------STIDKT      178
                               .  .        :     *.

Sc      TGRKSKS------KDMG------------LRSTALLAKMKKDRDDKETSRQNELRKRKEE      193
Hs      KETKTEAKEVTNASAAGNKAPGKGNMMSNFFGKAAMNKFKVNL---DSEQAVKEEKIVEQ      265
Ct      AEKATLSAPAKKGSSAPKSAASSGGIMQAFSKAAAVSKAKKQTPALRSATPASVEESIPQ      278
Sp      DTRST---EKTKGKD-------------IFSNAR-------------NQKGNSSRKNKKA      209
            :        .               :                 .    . .:

Sc      NLQKIN-------KQNPER------------EAQMKELNNLFVEDDLDTEEVNGGSKPN-      233
Hs      PTVSVT--EPKLATPAGLKKSSKKAEPVKVLQKEKKRGKRVALSDDETKETENMRKKRRR      323
Ct      PLSDDGEDDEDMPQPKPSRS-----SAMKTKKQREEELRRMMESDDEEEKKKKKE--EEE      331
Sp      PLENHKEKEPLLPKEE---------KLSEQAKRERDDLKNIMQLEDESVSTTSV------      254
           .                           : . .  ..:   :*   .  .

Sc      --SPKETDSNDKDKNNDDLEDLLET--TA-----EDSLMDVPKIQQTKP----SETEHSK      280
Hs      IKLPESDSSEDE--VFPDSPGAYEAESPSPPPPPSPPLEPVPKTEPEPPSVKSSSGENKR      381
Ct      EEEEEEEESEHE--QLPAEEEPMAEEPKAPEPVK----------EEPAEIITATTNGRRR      379
Sp      ------HDSEDD--NLDSNNFQLEIGTEAKSAAP----------DEPQEIIKSVSGGKRR      296
               .*:..                :               :            . :

Sc      EPKSEEEPSSFIDEDGYIVTKRPATSTP--------PRKPSPV-------VKRALSSSKK      325
Hs      KRKRVLKSKTYLDGEGCIVTEKVYESESCTDSEEELNMKTSSVHRPPAMTVKKEPREERK      441
Ct      GKRKVLRKKQIMDEQGYLVTVTEPAWESFSEDEPPPPSKPKATSLAPATQAT--------      431
Sp      GKRKVKKYATTKDEEGFLVTKEEEVWESFSEDENISTGTSNVVRNKPTTVNIAT----KK      352
          :   .     * :* :**                  . . .

Sc      Q--ETPSSNKRLKKQGTLESFFKRRKAK      350
Hs      GPKKGTAALGKANRQVSITGFFQRK--      466
Ct      KP-----KKGGKGGQGSIMSFFAKK--      451
Sp      KN-----TAQSKPQQKSIMSFFGKK--      372
                      * :: .:* :*
```

Supplementary Figure S1: Multiple protein sequence aligment (generated using Clustal Ω version 1.2.4) of PolD3 orthologues from human (UniProt accession number Q15054), *S. cerevisiae* (P47110), *C. thermophilum* (G0S636) and *S. pombe* (P30261). The PIP motif is boxed with conserved residues shown in bold red text. The DPIM motif (Gray et al., 2004, BMC Mol. Biol. 5, 21) is boxed in blue; the globular N-terminal domain seen in cryo-EM and crystal structures of human and *S. cerevisiae* orthologues is shaded in grey. Abbreviations: Hs, human; Sc, *S. cerevisiae*; Ct, *C. thermophilum*; Sp, *S.pombe*.

```
Hs          MGIQGLAKLIADVAPSAIRENDIKSYFGRKVAIDASMSIYQFLIAVRQ-GGDVLQNEEGE      59
Sc          MGIKGLNAIISEHVPSAIRKSDIKSFFGRKVAIDASMSLYQFLIAVRQQDGGQLTNEAGE      60
Ct          MGIKHLFQLIKEEAPLSIREGEIKQHFGRKVAIDASMSIYSFLIAVRS-DGQQLMNDAGE      59
Sp          MGIKGLAQVLSEHAPASVKHNDIKNYFGRKVAIDASMSLYQFLIQVRSQDGQQLMNEQGE      60
            ***: *  :: : .* :::..:**..************:*.*** **. .*  * *: **

Hs          TTSHLMGMFYRTIRMMENGIKPVYVFDGKPPQLKSGELAKRSERRAEAEKQLQQAQAAGA      119
Sc          TTSHLMGMFYRTLRMIDNGIKPCYVFDGKPPDLKSHELTKRSSRRVETEKKLAEAT---T      117
Ct          TTSHLMGMFYRTLRMVDAGIKPLYVFDGKPPKLKSGELAKRFQRKQEAQEDLEEAKETGT      119
Sp          TTSHLMGMFYRTLRIVDNGIKPCFVFDGKPPTLKSGELAKRVARHQKAREDQEETKEVGT      120
            ************:*::: **** :******* *** **:**  *: ::.:.  ::    :

Hs          EQEVEKFTKRLVKVTKQHNDECKHLLSLMGIPYLDAPSEAEASCAALVKAGKVYAAATED      179
Sc          ELEKMKQERRLVKVSKEHNEEAQKLLGLMGIPYIIAPTEAEAQCAELAKKGKVYAAASED      177
Ct          AEDVEKFSRRTVRVTREHNAECQRLLKLMGIPYIIAPTEAEAQCAVLARAGKVYAAASED      179
Sp          AEMVDRFAKRTVKVTRQHNDEAKRLLELMGIPFVNAPCEAEAQCAALARSGKVYAAASED      180
                 :  :* *:*:::** *.::** *****:: ** ****.** *.: *******:**

Hs          MDCLTFGSPVLMRHLTASEAKKLPIQEFHLSRILQELGLNQEQFVDLCILLGSDYCESIR      239
Sc          MDTLCYRTPFLLRHLTFSEAKKEPIHEIDTELVLRGLDLTIEQFVDLCIMLGCDYCESIR      237
Ct          MDTLCFNTPILLRHLTFAEQRKEPIQEIHTDKVLEGLNMDRKQFVDLCILLGCDYLDPIP      239
Sp          MDTLCFQAPVLLRHLTFSEQRKEPISEYNIEKALNGLDMSVEQFVDLCILLGCDYCEPIR      240
            ** * : :*.*:**** :* :* ** * . .  *. *.:  :*******:**.** : *

Hs          GIGPKRAVDLIQKHKSIEEIVRRL-----DPNKYPVPENWLHKEAHQLFLEPEVLDP--E      292
Sc          GVGPVTALKLIKTHGSIEKIVEFIESGESNNTKWKIPEDWPYKQARMLFLDPEVIDG--N      295
Ct          KIGPSTALKLIREHGSLEKVVEFIQND--PKKRYTIPEDWPYQDARELFFNPDVRQADDP      297
Sp          GVGPARAVELIRQYGTLDR---FVKEA--DRSKYPIPEDWPYEDARRLFLDAEVLPG--E      293
             :**  *:.**: : :::.    :       .:: :**:* :::*: **:: :*

Hs          SVELKWSEPNEEELIKFMCGEKQFSEERIRSGVKRLSKSRQGSTQGRLDDFFKVTGSLSS      352
Sc          EINLKWSPPKEKELIEYLCDDKKFSEERVKSGISRLKKGLKSGIQGRLDGFFQVVPKTKE      355
Ct          ECDFKWEKPDVEGLVQFLVKEKGFSEDRVRNGAQRLEKNLKGAQQARIEGFFKVIPKTEA      357
Sp          EIELKWKSPDADGIIQFLVKEKGFNEDRVKLGINRLEKASKTIPQGRLDSFFKPVPSSPK      353
            . ::**. *. . :::::  :* *.*:*:: * .**.*  :   *.*::.**:   .

Hs          AKRKEPEPKGSTKKKAKTGAAGKF--KRGK---------      380
Sc          QLAA-AAKRAQENKKLNKNKNKVTKGRR-----------      382
Ct          EKQA-QKRKIEEQIEARKKKAKEEKKEKAKQKAKPRGAA      395
Sp          KPVD-TKSKGSAKRKRDSNKGGESKKKR-----------      380
```

Supplementary Figure S2: Multiple protein sequence aligment (generated using Clustal Ω version 1.2.4) of Fen1 orthologues from human (UniProt accession number P39748), *S. cerevisiae* (P26793), *C. thermophilum* (G0S2B5) and *S. pombe* (P39750). The PIP motif is boxed with conserved residues shown in bold red text. Abbreviations: Hs, human; Sc, *S. cerevisiae*; Ct, *C. thermophilum*; Sp, *S.pombe*.

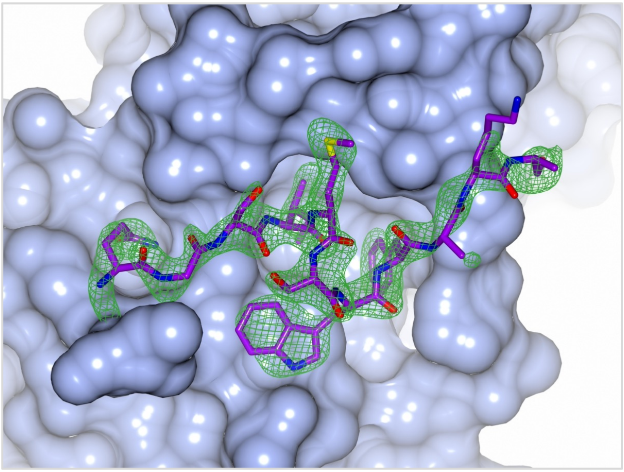

Ct PolD3 PIP peptide: ^{441}QGSIMSWFAKK^{551}

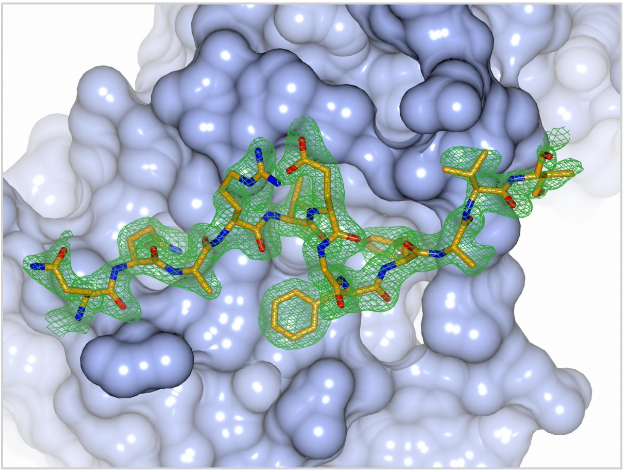

Ct Fen1 PIP peptide: ^{341}QQARIEGFFKVI^{352}

Supplementary Figure S3: Fo−Fc difference density maps calculated (ligand removed, model re-refined) for the *Ct* PolD3 (**upper panel**) and *Ct* Fen1 (**lower panel**) PIP peptides bound to *Ct* PCNA. The difference electron density, depicted as green mesh, is contoured at 2.5 σ.

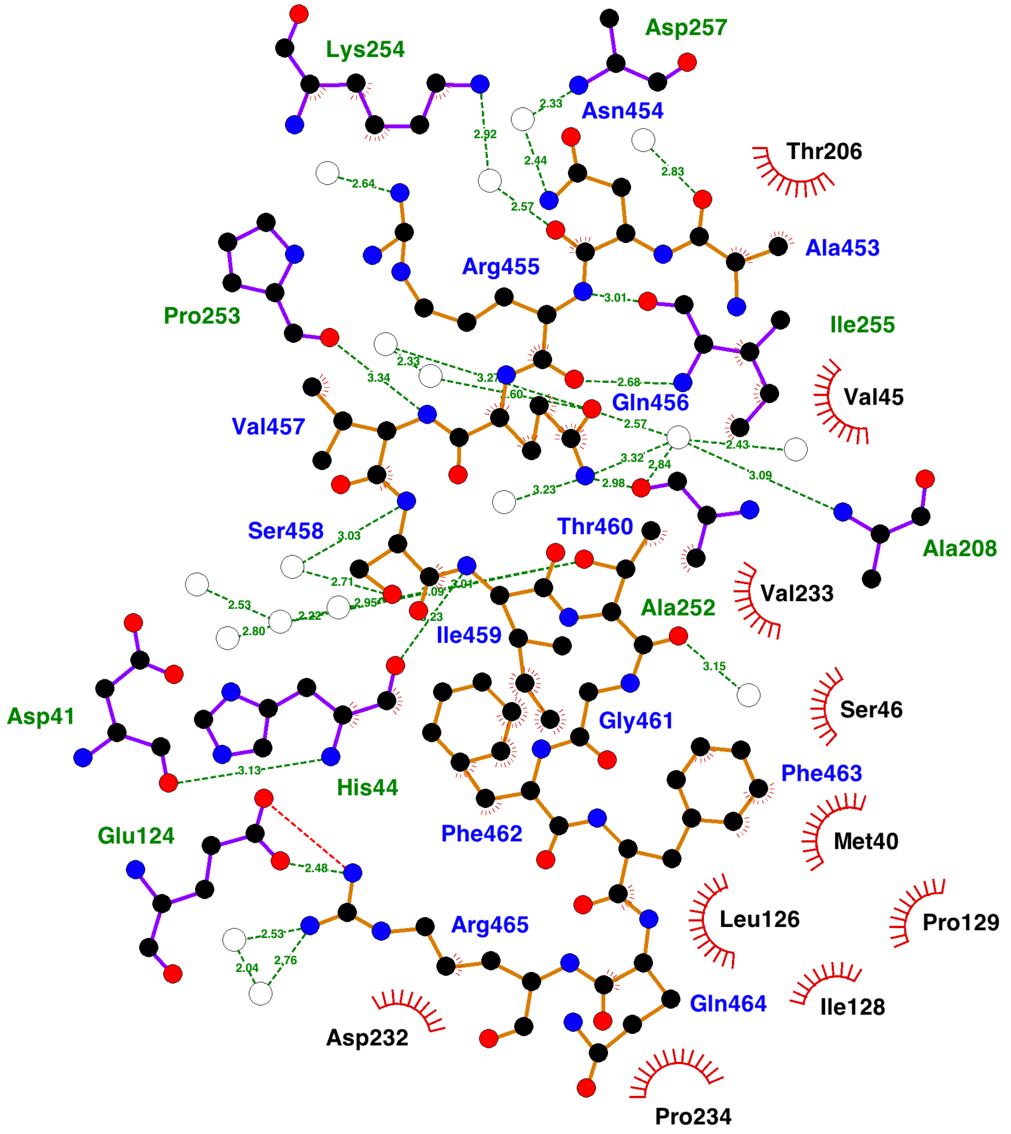

Supplementary Figure S4: LigPlot+ representation (Laskowski RA and Swindells MB, J. Chem. Inf. Model 51, 2778-86, 2011) of human PCNA–human p66/PolD3 PIP peptide interaction (PDB 1U76, described in Bruning JB and Shamoo Y, Structure 12, 2209-2219, 2004). The structure was solved to a resolution of 2.6Å.

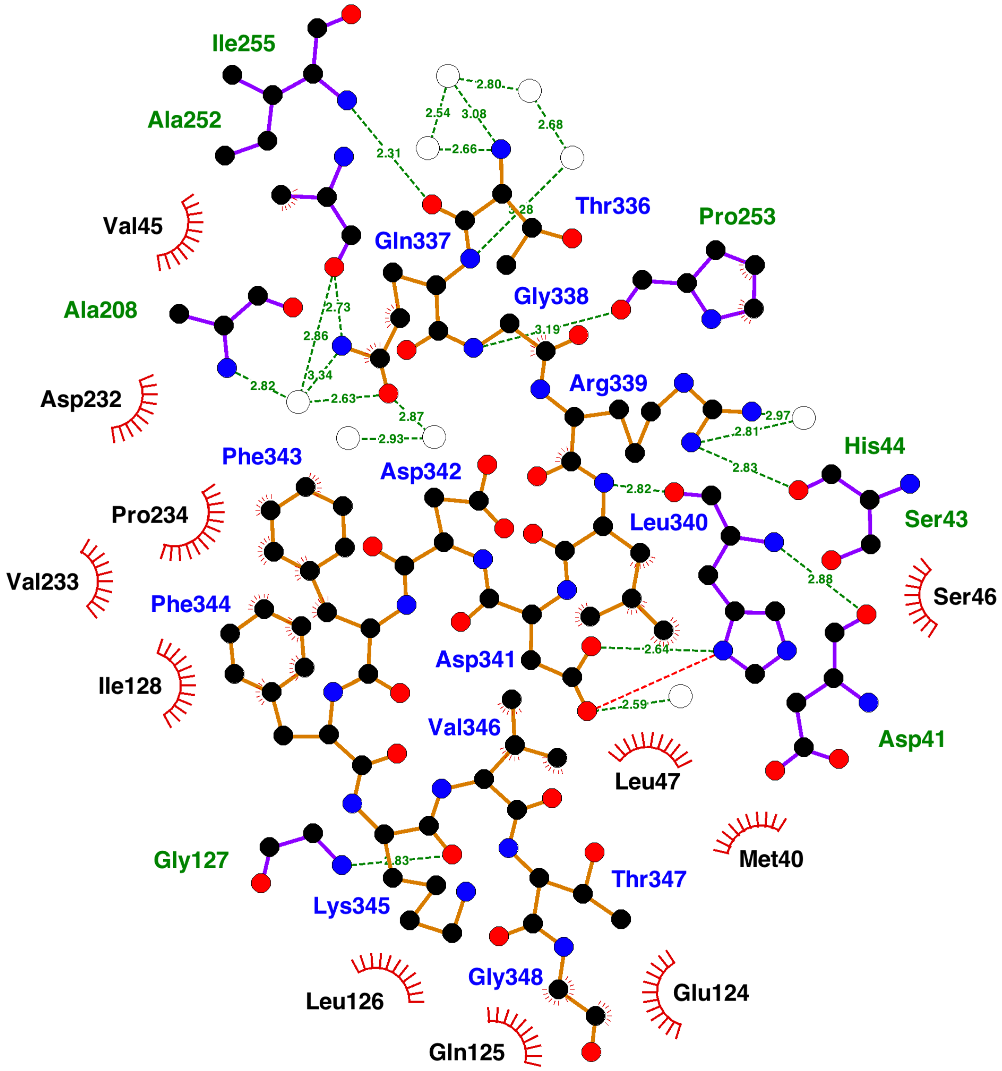

Supplementary Figure S5: LigPlot+ representation (Laskowski RA and Swindells MB, J. Chem. Inf. Model 51, 2778-86, 2011) of human PCNA–human Fen1 PIP peptide interaction (PDB 1U7B, described in Bruning JB and Shamoo Y, Structure 12, 2209-2219, 2004). The structure was solved to a resolution of 1.88 Å.
